# Tackling ciliary specialization to understand phenotypic variability in human primary ciliopathies

**DOI:** 10.1242/jcs.264177

**Published:** 2025-10-27

**Authors:** Ruxandra Bachmann-Gagescu, John A. Sayer

**Affiliations:** ^1^ Department of Molecular Life Sciences, University of Zurich, 8057 Zurich, Switzerland; ^2^ Institute of Medical Genetics, University of Zurich, 8952 Schlieren, Switzerland; ^3^ University Research Priority Program AdaBD, University of Zurich, 8057 Zurich, Switzerland; ^4^ Zurich Neuroscience Centre, University of Zurich, 8057 Zurich, Switzerland; ^5^ Biosciences Institute, Faculty of Medical Sciences, Newcastle University, Central Parkway, Newcastle upon Tyne, NE1 3BZ, UK; ^6^ Renal Services, Newcastle Upon Tyne Hospitals NHS Foundation Trust, Newcastle upon Tyne, NE7 7DN, UK; ^7^ NIHR Newcastle Biomedical Research Centre, Newcastle upon Tyne, NE4 5PL, UK; ^8^ Boston's Children's Hospital, Harvard Medical School, Boston, MA 02115, USA

**Keywords:** Primary cilia, Phenotypic heterogeneity, Cell-type-specific ciliary specialization, Fibro-cystic kidney disease, Retinal dystrophies, Neurodevelopmental ciliopathies, Multi-omics, iPSC-derived models, Organoids

## Abstract

Primary cilia are crucial cellular organelles with vital roles in signal transduction and cellular function. Disruptions in primary ciliary structure or function underlie a group of genetic disorders known as primary ciliopathies. These disorders present as a diverse range of clinical features with prominent phenotypic variability, often complicating their diagnosis and the basic understanding of their underlying molecular mechanisms. To grasp this complexity, the ciliopathy field is moving from a static view of primary cilia towards a more comprehensive understanding of their dynamic and specialized cell-type-specific roles. By building on the large amount of knowledge gathered over the past decades and by employing recently developed tools, including multi-omics and human cell-based *in vitro* models, we can now interrogate ciliary specialization to understand the role of cilia in each tissue and the consequences of ciliary gene dysfunction on human health. This Perspective explores the current challenges and opportunities associated with these modern tools and databases, highlighting important action points to advance our understanding of this fascinating organelle and its role in human health and disease.

## Teaser

**Summary:** Primary cilia are sensory organelles whose dysfunction causes ciliopathies. We explore how ciliary specialization might explain the high variability of ciliopathies and propose priority research directions.

## Introduction

Primary cilia have emerged as central regulators of cellular signaling and tissue homeostasis, and understanding their dynamic, cell-type-specific functions is key to unraveling the molecular complexity of ciliopathies.

### Recognition of the link between cilia and disease – coining the term ‘ciliopathies’

**Fig. 1.**
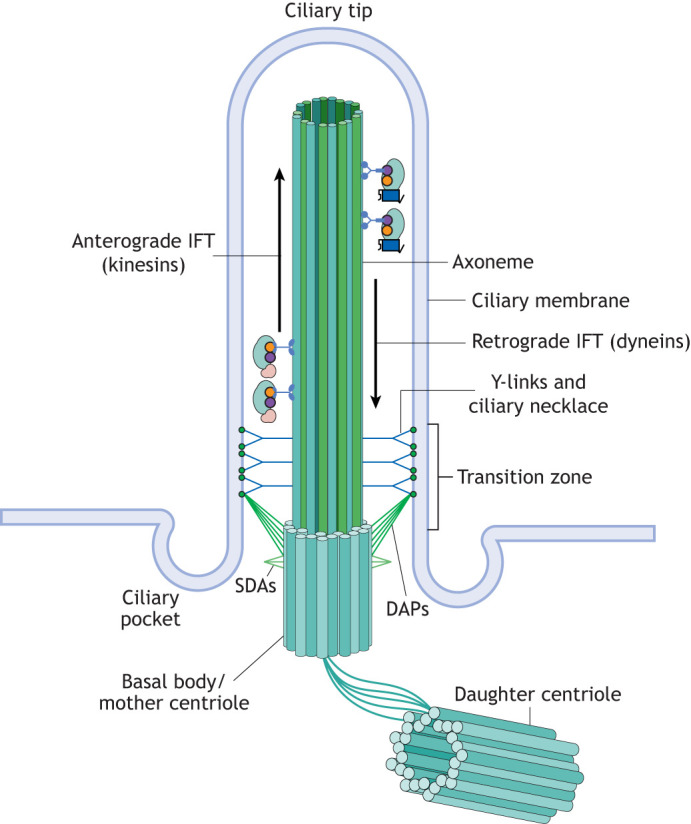
**The conserved structure of primary cilia.** The conserved structure of primary cilia includes different subcompartments – the basal body, which is a modified centriole; the transition zone (TZ), acting as gatekeeper to the ciliary compartment; the ciliary shaft whose core is formed by the microtubule doublets of the axoneme; and the ciliary tip. Distal (DAP) and subdistal (SDP) appendage proteins extend from the basal body, while Y-links join the TZ to the ciliary membrane. Movement of ciliary membrane proteins inside the cilium occurs through intraflagellar transport (IFT) from the base to the tip (anterograde, using kinesin motors) and from the tip to the base (retrograde, using dynein motors). The ciliary pocket is a specialized membrane domain at the base of the primary cilium, where the ciliary membrane is continuous with the plasma membrane but dips inward to form a pocket-like invagination.

Primary cilia are highly conserved throughout evolution. These typically rod-shaped structures are always composed of a microtubule-based axoneme anchored inside a modified centriole (the basal body) and are covered by a membrane that is continuous with the adjacent plasma membrane (Fig. 1). Previously considered vestigial, primary cilia were thought to have little functional significance (Anderson and Stearns, 2007). However, ∼20 years ago, the connection between cilia and human disease began to be recognized (Murcia et al., 2000). Revolutionary breakthroughs in the early 2000s have revealed that primary cilia play crucial roles in cellular signalling, development and organ function (Veland et al., 2009; Mill et al., 2023; Singla and Reiter, 2006). This led to the recognition of a new group of disorders termed ‘ciliopathies’, which are unified by underlying ciliary dysfunction (Badano et al., 2006). Despite their shared link to primary cilia, human ciliopathies are highly heterogenous with respect to their clinical features and can affect most organ systems, including most significantly the kidney, retina and brain (Brown and Witman, 2014; Devlin et al., 2025; Bachmann-Gagescu and Neuhauss, 2019; Valente et al., 2014). Although some typical primary ciliopathy features are purely developmental, such as polydactyly (the presence of extra digits) or encephalocele (a herniation of the brain resulting from a neural tube defect), others, such as fibro-cystic renal disease or retinal degeneration, are progressive.

### The multiple roles of primary cilia – important landmarks from the past 20 years

The majority of primary ciliopathies had previously been described clinically, but the identification of their underlying molecular genetic causes was boosted by the quantum leap in next-generation sequencing (NGS) technology in the wake of the human genome project (Wheway and Mitchison, 2019). The realization that the proteins encoded by these genes localize to the primary cilium (or the basal body) demonstrated that clinically distinct disorders, such as Bardet–Biedl syndrome (BBS), Joubert syndrome (JBTS), polycystic kidney disease (PKD) or Leber congenital amaurosis, share a pathophysiological link to this quasi-ubiquitous organelle (Cardenas-Rodriguez and Badano, 2009). Through highly collaborative efforts, the very productive cilia research community has gained substantial insights into the genetic makeup of ciliopathies and identified key roles for primary cilia in a multitude of processes. Today, a molecular diagnosis can be achieved in ∼60–70% of individuals with a primary ciliopathy disorder (on average), confirming the clinical diagnosis and allowing for genetic counselling, family planning and disease management (Bachmann-Gagescu et al., 2020; Dollfus et al., 2024). Large proteomics studies have identified several multiprotein complexes composed of multiple ciliopathy proteins that are localized to various ciliary sub-compartments (Boldt et al., 2016; van Dam et al., 2019; Garcia-Gonzalo et al., 2011; Sang et al., 2011; Latour et al., 2020; Taschner et al., 2016; Klena and Pigino, 2022). Primary cilia have been shown to transduce and regulate key developmental pathways, most prominently Hedgehog (Hh) (Goetz et al., 2009; Corbit et al., 2005), Wnt (Wheway et al., 2018; Lee, 2020), platelet-derived growth factor subunit A (PDGFRa) signalling (Christensen et al., 2017) and others, thereby regulating embryonic development through control of patterning, proliferation or differentiation. Furthermore, sensation of light and olfaction require functional cilia, the presence of cilia is linked to cell cycle control, and ciliary proteins have been suggested to act in DNA damage repair regulation pathways (Chaki et al., 2012; Kasahara and Inagaki, 2021; Bachmann-Gagescu and Neuhauss, 2019; Takeuchi, 2024). In fact, this multitude of highly diverse functions begs the question of how such a relatively small and simple structure can perform such a variety of tasks. In this Perspective, we will start by listing some of the challenges we currently face, in particular linked to the prominent phenotypic variability of ciliopathies, before discussing how ciliary specialization might provide some answers. We will focus on the opportunities offered by modern human cell-based *in vitro* models, omics approaches, and advanced imaging and bioinformatics to investigate ciliary specialization, while also mentioning current limitations and proposing concrete action points to advance the field to ultimately achieve improved management and treatment for individuals with ciliopathies.

## Current challenges in ciliopathy research – what do we need to learn next?

### Genetic diagnosis

Despite the tremendous progress achieved by the ciliopathy research community in the past two decades, many crucial questions remain open. From a clinical perspective, no monogenic cause can be identified in up to one third of individuals with a presumed primary ciliopathy syndrome (Shaheen et al., 2016; Bachmann-Gagescu et al., 2020). This is no different from other Mendelian disorders and is most likely explained by several factors. First, a few still unidentified gene–disease associations might exist, even though the number of remaining unrecognized clinically relevant ciliopathy genes is unlikely to be very large at this stage. Because current genetic diagnostic tests focus on variation in the coding regions of the genome, we expect that rare variants in non-coding regions (e.g. intronic or regulatory regions) probably account for many of the genetically unexplained cases, and we await breakthroughs in our ability to interpret such non-coding sequence variation. In parallel, substantial challenges also remain in interpreting the clinical significance of variants in coding regions, particularly for missense variants. Indeed, many diagnostic genetic tests yield so-called ‘variants of uncertain significance’ (VUS), which cannot be unequivocally classified as either benign or as pathogenic despite substantial progress in computational prediction algorithms (Vintschger et al., 2023; Chen et al., 2023; Katsonis et al., 2022).

### Predicting clinical outcome

Beyond identifying the precise genetic cause in a given individual with ciliopathy, even in those with a secured molecular diagnosis, the perhaps more daunting challenge is predicting the actual clinical consequences of the genetic findings. Primary ciliopathies are marked by prominent phenotypic variability, such that individuals with disease-causing variants in the same gene and sometimes even with the exact same genetic variants (as is the case for related individuals and founder alleles) will display quite drastically different clinical features (Focşa et al., 2021; Coppieters et al., 2010; Bachmann-Gagescu et al., 2015; Phelps et al., 2018). This is particularly problematic for predicting clinical outcomes in a pre-conceptional or prenatal setting and for determining the severity of progressive features such as retinal or renal disease. The reasons for this phenotypic variability remain unresolved and are most likely multifactorial, involving additional genetic variants (e.g. differences in genetic background and disease-modifying alleles), environmental factors and possibly pure stochasticity. Another possible explanation lies in cell-type-specific roles of primary cilia, which could explain why different disease-causing variants in the same gene can affect tissues differently.

### Understanding ciliary specialization

The multiplicity of primary cilia functions undoubtedly requires a high degree of specialization to respond to the stimuli relevant to each particular cell type. However, our current knowledge of ciliary biology mostly stems from cell culture systems or simple organisms that often lack diverse cilia types, such as the flagella in *Chlamydomonas* or the cilia of sensory neurons in *Caenorhabditis elegans* (Marshall, 2024; Blacque and Sanders, 2014; Ostrowski et al., 2011). As these models are more amenable to manipulations, they have been instrumental in studying ciliary biology, and are likely to yield many important future insights. Nevertheless, we will also need to tackle the question of cell-type specificity of primary cilia to fully understand the phenotypic variability observed in individuals with ciliopathies and predict clinical outcomes more precisely. More complex animal models, such as mouse and zebrafish, undeniably remain essential and could help address some fundamental aspects of ciliary specialization as well as putative varying roles of primary cilia across time and place (Rusterholz et al., 2022; Rao Damerla et al., 2014). In fact, even in *Caenorhabditis elegans*, clear distinctions between cilia of different sensory neurons have already been described, demonstrating ciliary specialization even in this ‘simple’ organism (Mukhopadhyay et al., 2007). However, the very idea that any given cell type has its own type of cilium will also require directly studying cilia in relevant human cells. This is now becoming possible as a result of new human cell-based *in vitro* models.

### Understanding organ-specific pathomechanisms underlying human ciliopathies

The question of ciliary specialization contains a corollary in the context of human disease – can a given cilium dysfunction in several independent distinct ways or is it a matter of the degree of dysfunction of a specific predominant role for any particular cilium type? In other words, is phenotypic variability explained by different levels or by different ‘flavours’ of ciliary dysfunction? Fundamentally, addressing this question requires determining whether a given primary cilium simultaneously transduces and regulates multiple signals (using separate pathways that might or might not influence each other) or whether each cilium mostly handles one sensory-signalling function at a time. The answers to these questions will in part determine whether dysfunction of distinct ciliary proteins can result in different effects in each tissue. Evidence supporting the hypothesis that ciliary dysfunction is more complex than a simple spectrum of severity comes precisely from individuals with ciliopathies; biallelic mutations in *CEP290,* for instance, can result in isolated severe retinal disease in the form of Leber congenital amaurosis, isolated severe renal disease in the form of nephronophthisis or a multisystemic ciliopathy syndrome in the form of JBTS, which is defined by presence of a central nervous system (CNS) malformation (Coppieters et al., 2010). Hence, in the same individual, one organ might exhibit primary cilia dysfunction whereas in other organs, ciliary function is either sufficient or redundant enough to preserve a normal phenotype. To tackle these questions, the field is moving from a static view of primary cilia in simple systems towards a more differentiated understanding of the dynamic and context-dependent roles of primary cilia in different tissues, places and times.

Last but not least, identifying therapeutic targets for primary ciliopathies – the most important expectation individuals with ciliopathies have for our community – will require understanding the molecular pathomechanisms in a given tissue. From a clinical perspective, advancing ciliopathy research to this next level will require more precise prediction of clinical outcome to identify which of the symptoms an individual has will progress over time and require treatment. When combined, accomplishing these goals will enable the targeted development and delivery of such treatments. For both aspects, understanding the dynamic behaviour of primary cilia and defining their cell-type-specific adaptations are crucial next steps, in which new model systems derived from human individuals with pathogenic variants will play a key role.

## Addressing ciliary specialization in human cells – possibilities and current limitations of new cellular models

**Fig. 2.**
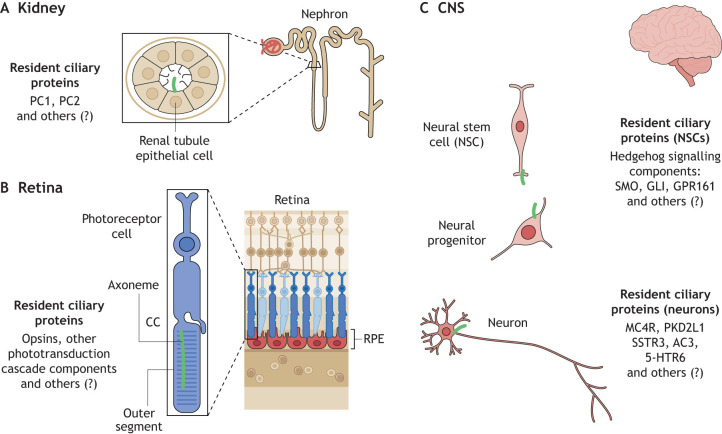
**Organ and cell-type-specific functions and pathways of primary cilia.** There is a need to continue to define the dynamic and cell-type-specific sensory functions of cilia. Here, we show examples of renal, retinal and CNS ciliated cells, indicating some of the known resident ciliary proteins required for their specific sensory function. (A) Within the kidney, renal tubular epithelial cells express PC1 and PC2 on their primary cilium and are crucial for cell polarity and orientation. (B) Within photoreceptor cells, which have evolved a highly specialized ciliary compartment called the outer segment (OS), which is linked to the cell body through the TZ-equivalent connecting cilium (CC), opsins and other phototransduction cascade components are required for light sensation as a very special modality of ciliary signalling. (C) Within the CNS, neural stem cells and neural progenitor cells respond to Hh signalling through pathway components Smoothened (SMO), GLIs and GP161. Neurons have known G protein-coupled receptors including melanocortin 4 receptor (MC4R), polycystin-2-like 1 (PKD2L1), somatostatin receptor 3 (SSTR3), adenylate cyclase 3 (AC3) or serotonin receptor 5-hydroxytrptamine receptor 6 (5-HTR6). In each tissue (and cell) other ciliary signalling pathways (known and unknown) are also involved denoted by ‘others (?)’.

Although primary cilia specialization is evident, its extent has not yet been systematically addressed. Primary cilia vary morphologically between tissues (Jurisch-Yaksi et al., 2024; Mill et al., 2023), most strikingly evidenced by the photoreceptor cilium, which has evolved into the so-called outer segment (see below and Fig. 2) (Bachmann-Gagescu and Neuhauss, 2019). In most other human tissues, cilia remain rod-like, but their length varies substantially and dynamically. Additionally, though the ciliary membrane composition must vary across cilia types with respect to G protein-coupled receptors (GPCRs) (Mykytyn and Askwith, 2017) and other sensing proteins, to accommodate the specialized signalling functions of a particular cell (Fig. 2 and Table 1), it remains unclear how this is precisely achieved. The transition zone (TZ) complexes control ciliary content by acting as a gatekeeper for entry and exit to the ciliary compartment (Garcia-Gonzalo and Reiter, 2017), but it is currently unknown whether they are identical in all cilia types. Similarly, we do not know whether all ciliopathy proteins are always expressed together in all cilium types and whether they always localize to the same ciliary sub-compartment and interact within the same protein complexes. For example, CEP290 has been reported to localize at the centriolar satellites, the centrosome and the TZ, but these apparently conflicting reports might in fact be easily explained by cell-type-specific roles for CEP290 (Sayer et al., 2006; Craige et al., 2010; Mercey et al., 2024; Kim et al., 2008). Even within the same cell type, a particular function and localization for a protein might vary according to cell state. The following sections will discuss the use of human cellular models to explore the cell-type-specific roles of ciliopathy proteins in three cell types that are highly relevant in primary ciliopathy disease – renal tubular cells, retinal photoreceptors and neurons.

**Table 1.** An overview of key unknowns for primary cilia in kidney, retina and CNS

	CNS	Retina	Kidney
**Main cell type studied in the context of ciliopathies so far**	Neural stem cells	Photoreceptors	Tubular epithelial cells
**Main role(s) assigned to cilia so far**	Sensation of morphogens during development	Light sensation	Mechanosensation (?)
**Key unknowns**			
Type of signalling	Role of primary cilia in differentiated neurons – through direct contacts? – neuromodulation? – regulation of synapses and/or synaptic function?	Do photoreceptors have a sensory role beyond light perception and do they interact with other retinal cell types via signalling pathways?	Relative importance of sensation of chemical (?) versus mechanical (e.g. fluid flow) stimuli?
Mechanistic aspects	Evolving requirement for ciliary signalling throughout neural development: – proliferation versus differentiation? – timings of cilia assembly, disassembly?	Exact molecular mechanism of protein (opsin) transport – passage through the connecting cilium?	Precise molecular mechanism by which renal cilia sense and transduce mechanical stimuli into biochemical signals
Pathomechanisms	How do ciliary defects lead to specific cortical and cerebellar malformations?	What explains the progression of the retinal dystrophy and can we halt it?	What distinguishes causative pathogenic versus adaptive ciliary signalling responses?
Role of other cell types	Role of cilia on astrocytes?	Role of ciliary defects on non-photoreceptor cells (e.g. RPE cells)?	Are there nephron region-specific ciliary functions in proximal versus distal tubules and role of cilia on non-tubular cells (e.g. interstitial fibroblasts)?

### Renal tubular cells

In the kidney, the archetypal primary ciliopathy disease is nephronophthisis, defined by fibrotic changes predominantly occurring around the distal convoluted tubule, a region that experiences high exposure to genotoxins (Srivastava et al., 2017). Primary cilia dynamically change their length and structure in response to kidney injuries, implying a tissue remodelling component to their function and the existence of dynamic processes that enable renal tubular cells to respond to damage and regenerative signals. The precise link between ciliary dysfunction and interstitial fibrosis, however, remains unclear.

Renal ciliopathies are also associated with cystic kidney disease phenotypes, highlighted by autosomal dominant polycystic kidney disease (ADPKD), associated with pathological variants in polycystin-1 (PC1, encoded by *PKD1*) and polycystin-2 (PC2, encoded by *PKD2*) (Chebib et al., 2025; Bergmann et al., 2018; Orr and Sayer, 2023). In ADPKD, cysts are thought to result from defects in cilia-related proteins that disrupt sensing, signalling, polarity and/or differentiation in renal tubule cells. These deficiencies gradually turn narrow renal tubules into fluid-filled cysts. Proposed mechanisms, which remain controversial, include loss of flow mechanosensation by the primary cilium leading to aberrant Ca^2+^ and cAMP signalling, uncontrolled cell proliferation and cyst formation (Nauli et al., 2003; Delling et al., 2016). Whether disrupted planar cell polarity potentially contributes mechanistically to cyst formation has also been debated, as cilia are key for proper alignment of cells within a tissue plane (Nigro et al., 2015; Wallingford, 2012; Kunimoto et al., 2017). Defective cilia might also lead to misoriented cell division, leading to dilated tubules that promote cyst initiation (Jonassen et al., 2008). Many ciliary proteins, including PC1 and PC2 (Fig. 2), are essential for maintaining tubule architecture and function (Gilder et al., 2018; Walker et al., 2019). Mutations in the genes encoding these proteins impair their ciliary localization and their ability to perform essential signalling. However, given the multiple and perhaps parallel explanations for cystogenesis, it remains to be determined which is the predominant pathomechanism, an important consideration for future therapies.

A powerful strategy to understand primary ciliopathies and their underlying mechanisms is the use of cells derived from individuals with ciliopathies as *in vitro* models. Urine-derived renal epithelia cells (URECs) can provide a robust individual-specific model of renal tubular epithelia. URECs reliably form primary cilia, making them ideal for *in vitro* cilia studies (Molinari and Sayer, 2019; Molinari et al., 2020; Dewhurst et al., 2021). They can be used to observe cilia assembly, disassembly, signalling pathways and cilia-dependent mechanotransduction, and can also be manipulated in 2D and 3D culture systems to help study how specific mutations affect ciliary morphology and how this contributes to disease manifestation (Hynes et al., 2014). Downstream applications useful for deep phenotypic analysis include spheroids, tubuloids, organoids and ‘kidney-on-a chip’ systems (Nishimura, 2024; Forbes et al., 2018).

Another source of such derived cells for *in vitro* modelling are induced pluripotent stem cells (iPSCs), which can be reprogrammed from skin fibroblasts or peripheral blood mononuclear cells very efficiently. A variety of differentiation protocols have been published that allow generation of renal organoids containing most segments of the nephron (Takasato et al., 2016; Morizane et al., 2017). Such models can be used to study disease mechanisms, taking into account the genetic makeup of affected individuals and represent promising models for drug screening.

However, such *in vitro* models have limitations. It remains particularly challenging to reproduce the complex tissue microarchitecture or the interplay between different cell types, especially if cells arise from different germ layers, as these typically require distinct differentiation factors. For example, kidney organoids lack immune cells and vascular perfusion (Dorison et al., 2022). Consequently, there is no glomerular filtration and thus filtrate to generate flow within tubules. Approaches to mitigate these limitations include transplantation into an animal and co-culture protocols (Bas-Cristóbal Menéndez et al., 2022). Additional limitations include ciliary heterogeneity, which is hard to control within these model systems, and variability in differentiation protocols between laboratories, which generates datasets that are difficult to compare.

### Retinal photoreceptors

The cilia of photoreceptors (PRs), are the most striking example of cell-type-specific ciliary specialization. To accommodate the massive quantity of proteins required for the phototransduction cascade and allow light perception, PRs have massively expanded the surface area of their ciliary membrane by building stacks of membrane disks or folds, forming the so-called outer segments (OSs) (Fig. 2) (Masek and Bachmann-Gagescu, 2023). The OS is linked to the cell body, specifically to the inner segment, which also contains other organelles, such as mitochondria or the Golgi, through the connecting cilium (CC). These membrane stacks are regularly replenished from the OS base and phagocytosed from the tip by the retinal pigment epithelium (RPE) (May-Simera et al., 2018, 2017). Because the major components of the OS are recycled through this mechanism, there is likely less need for ciliary retrograde trafficking from this compartment back to the cell body. Nevertheless, functional intraflagellar transport (IFT) in both directions is required, because mutations in genes encoding both anterograde and retrograde IFT components lead to retinal dystrophy (Gupta and Pazour, 2024; Insinna and Besharse, 2008). Vast amounts of protein must continuously traffic towards the OS and through the narrow CC, implying the presence of highly developed vesicle trafficking pathways and elaborate sorting mechanisms, which might be highly sensitive to disruptions (Sánchez-Bellver et al., 2021; Ojeda Naharros et al., 2017). The CC of a photoreceptor cell is considered equivalent to the TZ of other cilia, but it contains specific proteins that are not present in other cilia, such as SPATA7, which marks another specialized photoreceptor-specific sub-ciliary compartment (Dharmat et al., 2018). Similarly, the presence of lebercilin (LCA5) at the apical end of the CC marks a region somewhat functionally equivalent to the ciliary tip, even though axonemal microtubules extend above the CC into the actual outer segment (Faber et al., 2023).

Mouse and zebrafish models have been instrumental in understanding the molecular underpinnings of the photoreceptor primary cilium, as the retina is particularly well conserved across species (Masek et al., 2023; Collin et al., 2020; Perkins, 2022). However, notable differences exist, such as the paucity of cones in the mouse retina or the capacity for regeneration of the zebrafish retina, which impact modelling of retinal degeneration to some extent (Wan and Goldman, 2016; Masek and Bachmann-Gagescu, 2023). Human iPSC-derived retinal organoids represent a promising new avenue to investigate the role of ciliopathy proteins in human photoreceptors, including for personalized disease modelling using cells derived from individuals with ciliopathies (Afanasyeva et al., 2021; Lee and Jo, 2025). Current challenges in this field are the long timelines of currently available protocols – typically requiring >150 days to generate differentiated photoreceptors – which limit the scalability of such systems for investigating the tissue-specific effects of genetic variants identified in individuals with ciliopathy or for drug testing approaches. Another important current limitation of retinal organoids is the absence of RPE cells and thus their supportive role in photoreceptor homeostasis. Given their crucial role in phagocytosis of OSs, recycling of phototransduction cascade components, production of trophic factors necessary to PRs and structural support (Wang et al., 2024), the absence of RPE cells precludes development of fully functional photoreceptors, illustrating how the complex interplay of different cell types in the normal tissue microarchitecture are still challenging to reproduce *in vitro*. However, given the progress in the field, including the development of ‘retina-on-a-chip’ systems composed of the combination of initially independently grown retinal organoids and RPE (Achberger et al., 2019), we anticipate improvements on this front towards more physiologically relevant models.

### Neural stem cells and neurons

The importance of primary cilia in the CNS is underscored by the prototypical primary ciliopathy JBTS, which is characterized by a pathognomonic cerebellar and brain stem malformation termed ‘the molar tooth sign’ (MTS) due to its appearance on axial brain MRI (Maria et al., 1997). Individuals with JBTS can also present with additional supratentorial anomalies including ventriculomegaly, nodular heterotopias (atypically localized foci of grey matter) or agenesis of the corpus callosum, among others (Bachmann-Gagescu et al., 2015, 2020; Poretti et al., 2017). Beyond JBTS, CNS involvement is also found in other ciliopathies, in particular in BBS, in which many affected individuals present with learning disabilities and sometimes seizures despite the lack of obvious morphological anomalies upon brain imaging (Forsythe and Beales, 2013; Baker et al., 2011). In fact, even obesity, which is a major phenotype in BBS, is caused by aberrant ciliary signalling in the CNS through the melanocortin receptor MCR4 in the hypothalamus leading to hyperphagia (Wang et al., 2021). As therapies targeting this receptor are now becoming available, this perfectly illustrates how strategies focusing on specialized ciliary functions can have beneficial effects for affected individuals (Argente et al., 2025).

Primary cilia function in the developing nervous system has been abundantly studied in various animal models, which has aided in identifying roles in patterning of the early neural tube, control of cell proliferation, migration and axon pathfinding (Spassky et al., 2008; Goetz and Anderson, 2010; Baudoin et al., 2012; Dumoulin et al., 2024; Higginbotham et al., 2013, 2012; Guo et al., 2015, 2019). Many of these roles rely on sensation of secreted molecules, mostly morphogens, from signalling pathways, in particular Hh signalling (Goetz et al., 2009; Corbit et al., 2005) (Fig. 2). Primary cilia on early neuronal cells therefore appear to mostly fulfil the canonical signalling function of cilia by binding secreted ligands. This role of primary cilia during CNS development likely explains the MTS and other morphological changes, such as nodular heterotopias (caused by migration defects) or agenesis of the corpus callosum (caused by axonal pathfinding defects) (Stoufflet and Caillé, 2022; Bachmann-Gagescu et al., 2020; Dumoulin et al., 2024).

More recent work focusing on the mature CNS now suggests different roles for primary cilia on mature neurons that rely on direct contact or close proximity between cilia and other cells or cellular structures rather than on sensation of secreted morphogens. For example, axons from serotonergic brainstem neurons have been shown to form synapses on cilia of hippocampal CA1 pyramidal neurons of mice (Sheu et al., 2022). Recent studies using 3D reconstruction of serial transmission electron microscopy images from human cortex and mouse occipital cortex have demonstrated that cilia of mature neurons make contacts with a multitude of axons and neurites of other neurons and are ideally placed at the synapses between adjacent neurons to sense neurotransmitters in the synaptic cleft (Wu et al., 2024; Ott et al., 2024). These new findings strongly suggest that primary cilia on mature neurons might play very different roles from those of cilia in neuronal progenitors. A recent study that performed a systematic imaging-based assessment of primary cilia in iPSC-derived neuronal cells generated by a variety of protocols (Haenseler et al., 2025) highlighted that the density of the culture influenced the ciliation rate and that cilia on more mature neurons were less responsive to stimulation by Sonic Hedgehog (SHH) signalling. Both findings support a model in which the main role of primary cilia on maturing neurons switches from sensing secreted morphogens to different roles requiring direct interactions.

This illustrates how iPSC-based modelling opens exciting new perspectives to study such roles of cilia in human neurons, allowing us to generate a wide variety of neuron types *in vitro*. A multitude of protocols for differentiation in two-dimensional (2D) and three-dimensional (3D) culture platforms are now available, such that many neuron types can be produced efficiently (Mayhew and Singhania, 2023; Paşca et al., 2015; Zhang et al., 2013; Muguruma et al., 2015). One caveat is the trade-off between directed and undirected differentiation protocols; although the latter have the disadvantage of higher variability, the former rely on the use of small molecules that modulate developmental signalling pathways transduced by cilia (such as Hh and Wnt), which can impact the efficiency of the protocol and/or complicate the interpretation of the role of the ciliary gene of interest in the resulting cultures (Sheta et al., 2023). Limitations of iPSC-derived neurons in general include variability across cell lines and differentiation batches, long generation times required to obtain mature neurons and reduced complexity of interacting cell types, although all are currently being addressed with ever-improving protocols. Moreover, as neural circuits *in vivo* form and adapt in response to stimulations which are largely absent in *in vitro* systems, the lack of stimulation-dependent activity remains a limitation even in more complex 3D organoid models. The use of optogenetics and electrical stimulations can partially address this problem (Mansour et al., 2018; Passaro and Stice, 2021; Li et al., 2024), but we are still far from reproducing stimulations and functional circuits that closely resemble physiological conditions.

In summary, new cellular models provide powerful platforms to investigate the complex, context-specific roles of primary cilia in human health and disease. Although these models are advancing rapidly, they must be refined and standardized to fully capture the specialized functions of cilia across different cell types. The use of higher-throughput methods, including omics approaches (e.g. single-cell and spatial transcriptomics, and proximity-labelling proteomics) and high-content iterative indirect immunofluorescence imaging (4i) (Kramer et al., 2023), will partly compensate for variability between organoids by providing more global and reliable assessments. Other advanced imaging approaches [e.g. expansion microscopy (Mercey et al., 2022) and/or super-resolution microscopy and cryo-electron microscopy] can uncover detailed structural differences in cilia between tissues and disease states. Integration of organoids with microfluidic systems could improve physiological relevance by mimicking fluid flow and mechanical forces. Better recreation of the complex tissue microarchitecture and inclusion of all relevant cell types will also be crucial to understanding non-cell-autonomous parameters influencing ciliary specialization, such as mechanical forces from fluid flow, and the role of the extracellular matrix or paracrine signals from neighbouring (even non-ciliated) cells. Finally, machine learning and computational modelling could help to integrate all these levels of information and identify patterns linking genetics and ciliary biology.

## Tapping into big data

The parallel quantum leaps in omics technologies and high-throughput imaging, both enabled by massively increased computational power, have generated very large publicly available datasets. By leveraging new computational approaches, we can now build on this acquired knowledge to tackle some of the fundamental outstanding questions outlined above.

### Integrating different levels of information from diverse datasets

Community efforts have produced a variety of very large datasets that contain information about human genetic variation, RNA expression across tissues, protein interaction networks, protein structures, subcellular localization and functional annotations. Large-scale efforts of the cilia community have generated cilia-specific datasets [SYSCILIA (Vasquez et al., 2021; van Dam et al., 2019), CiliaCarta (Vasquez et al., 2021; van Dam et al., 2019)] and the human Protein Atlas (https://www.proteinatlas.org/) now includes immunofluorescence datasets for cilia-localized proteins (Hansen et al., 2025). Alphafold2 enables modelling of 3D protein structures, onto which human disease variants can be mapped (Yang et al., 2023), and steadily improving computational prediction algorithms support classification of genetic variants as pathogenic or benign (https://varsome.com/). Finally, the primary literature describing disease phenotypes and genetic variants, functional assays and biological experiments *in vivo* and *in vitro* is ever-increasing.

The challenge now lies in integrating all these levels of information to gain deeper insights into ciliary biology and disease. For example, mapping pathogenic variants onto 3D protein structures while integrating expression data for the respective gene with putative tissue-specific isoforms or protein interactions could improve our prediction of the phenotype for a given individual. Such multi-level integration necessitates advanced computational approaches, which will require close collaboration and a common language between bioinformaticians and clinicians in order to harness the full power and direct clinical utility of bioinformatics. Furthermore, gaining useful new insights through bioinformatics requires large high-quality datasets. Unfortunately, large transcriptomics and proteomics datasets often contain limited reliable information about ciliary genes and proteins, which are typically expressed at very low levels. Developmental datasets are also still underrepresented, even though cilia are particularly important during development. From a clinical point of view, the individual-based phenotypic information available in the literature remains very patchy and heterogeneous.

### Gaining biological insights from big data

With so much information now within reach, this wealth of data can hopefully begin to guide or inform basic biological research. So far, several projects have taken a primarily bioinformatics approach to identifying new ciliary or ciliopathy-related genes and to understanding ciliary biology. The cilia and centrosome complex interactome (CCCI) project employed a network-based approach to analyse interactions among ciliary and centrosomal proteins. By integrating data from multiple sources, the researchers identified 1695 ciliary genes and revealed functionally specialized communities, consisting of highly connected and co-expressed genes, within the interactome, such as those involved in mRNA processing, protein synthesis and degradation (Amato et al., 2014), providing insights into the molecular mechanisms underlying ciliopathies and suggesting potential therapeutic targets. A more recent study integrated data from comparative genomics, protein–protein interactions, single-cell RNA sequencing studies, transcription factor networks and text mining to develop a tool for predicting novel ciliary genes called CilioGenics (Pir et al., 2024). Another recent study applied a computational framework to analyse proteomic data from over 400 tandem affinity purification-mass spectrometry (TAP-MS) experiments, focusing on ciliary proteins (Boldt et al., 2016). The researchers identified known ciliary complexes and predicted novel ones, providing a comprehensive view of the ciliary protein interaction landscape, data which has improved our understanding of ciliary functions and their implications in health and disease.

In a systems biology approach based on network propagation, using an algorithm that spreads along networks of protein interaction information from databases starting from selected ciliopathy seed genes, we have recently attempted to identify ciliary submodules associated with organ-specific phenotypes (Aarts et al., 2025 preprint). With this method, we found both already known patterns (for instance, association of IFT genes with skeletal phenotypes) as well as some interesting new modules (for example, a module involved primarily in renal ciliopathies). However, the large phenotypic and genetic overlap between disorders and the loose assignment of genes with clinical phenotypes in existing databases used as starting points made it challenging to identify many additional strong genotype–phenotype associations. This network propagation approach – in particular, when combined with knowledge gained from mouse phenotype databases – has allowed us to prioritize candidate genes for human primary ciliopathies, leading to identification of the likely genetic cause in previously undiagnosed individuals with ciliopathy. Thus, a bioinformatics-first approach, relying on multiple datasets, can be employed to understand rare disorders like ciliopathies. An important limitation for such approaches, however, lies in the circularity problem – given that algorithms build on previous data they are more likely to find related results again. This caveat also once again stresses the importance of the reliability of the underlying databases.

## Future directions – where do we go from here?

Advancing our understanding of ciliopathies demands a coordinated, multidisciplinary effort spanning clinical medicine, genomics and multi-omics, and molecular and developmental biology, as well as computational biology (Table 2). To move forward, collaboration across disciplines, institutions and borders is essential. Given that ciliopathies are rare disorders, it is crucial for the community to join forces and pool clinical information in standardized ways, making the data accessible, comparable and collected in curated registries. Several large-scale consortia have emerged to start addressing this including the PKD-RRC (https://www.pkd-rrc.org/) and TheRaCiL (https://theracil.eu/).

**Table 2.** Priorities for primary ciliopathy research using human cell-based models, phenotyping and bioinformatics

In the lab:	In the clinic:	At the computer:
**Improve human cellular models** Standardize differentiation protocols Reduce batch variability Increase physiological relevance **Cerebral organoids** Stimulation-dependent circuit formation and function Reduce culture time **Retinal organoids** Incorporate RPE – achieve ultra-structurally complete OS Reduce culture time **Renal organoids** Incorporate vascularization Achieve filtration and tubular fluid flow Improve culture of urine derived epithelial cells **Define ciliary proteome in different cilium types** Conservation of known protein complexes across cilia? Catalogue GPCR variation in ciliary membrane **Generate cell-type-specific gene expression datasets** Identify unique or enriched ciliary genes in specific cell types	**Improve patient phenotypic and genetic information** Systematic evaluation of patient phenotypes Standardize phenotypic descriptions (HPO terms) Pool data from multiple cohorts – curated registries (ERNs) Improve curation of genetic variants (ClinVar) Investigate genotype and phenotype correlations in pooled cohorts Integrate rare and common variants per individual **Generate comprehensive genetic data on patients** WGS Transcriptome when affected tissues are available	**Improve databases with respect to ciliary genes/proteins** Tissue-specific gene expression databases (GTExPortal) Tissue-specific protein isoforms Protein expression and intracellular localization (HPA) Build on previous large-scale cilia-specific efforts (SYSCILIA) to improve protein interaction data Curate protein interaction databases (STRING, BioGRID) **Ciliary protein structures and complexes** Alphafold for predicting 3D protein structures and identifying interaction interphases Map patient gene variants onto 3D models Improve prediction of the effect of variants **Integrate knowledge from different levels or develop machine learning approaches** to prioritize candidate ciliopathy genes to gain new biological insights (to be tested experimentally) to predict phenotypic outcome in patients to identify candidate therapeutic targets

Action points to prioritize for advancing primary ciliopathy research in the lab, in the clinic and at the computer. Full URLs of the databases are: ALPHAFOLD, https://alphafold.com; BioGRID, https://thebiogrid.org, ClinVar, https://www.ncbi.nlm.nih.gov/clinvar/, ERN European Reference Networks, https://health.ec.europa.eu/rare-diseases-and-european-reference-networks/european-reference-networks_en; GTExPortal, https://www.gtexportal.org; HPO terms Human Phenotype Ontology, https://hpo.jax.org; STRING, https://string-db.org; SYSCILIA, https://syscilia.org/. WGS, whole-genome sequencing.

Even when pooled, ciliopathy cohorts will remain comparatively small with highly heterogenous genetic data. Hopefully, machine learning will be able to recognize patterns by integrating ‘real world’ clinical data from well-curated pooled ciliopathy cohorts with the vast amount of knowledge available in various databases. Integrating high-quality comprehensive clinical and genetic data from pooled ciliopathy cohorts with data from large genomics consortia that offer structured, longitudinal data collection from genetically diverse populations [e.g. the Genome Aggregation Database (GnomAD)], gene expression datasets [e.g. single-cell RNA sequencing studies and genotype-tissue expression data (GTEx)], reliable protein–protein interaction networks (particularly those that are experimentally determined, e.g. CiliaCarta) and other multi-omics datasets could decode the cellular heterogeneity of diverse cilia types, describe the pathomechanisms underlying the specific ciliopathy phenotypes and identify therapeutic targets. To reach this goal, high quality datasets of ciliopathy models, including cellular and organoid models derived from animals and cells from human individuals, will be crucial, as computational models built with machine learning are only as good as the data they rely on. Transcriptomic and proteomic databases must therefore be improved by taking developmental stages into account and enhancing the detection of ciliary genes, which are typically expressed at low levels. This will require generating additional multi-omics datasets based on human tissues, where available, or on cellular or organoid models using cells derived from human individuals. Concurrently, existing cellular and organoid models must be improved, with the aim of standardizing protocols, reducing variability across batches and cell lines and better reproducing complex tissue microarchitecture and physiological functions. In parallel, established animal models remain relevant for new discoveries, for validation of results from cellular models through functional studies at the organismal level and for testing therapies in whole organisms.

Together, the cilia and ciliopathy research community has started to tackle these challenges and is on the right path towards improving our understanding of ciliary biology, ciliopathy genetics, disease mechanisms of ciliopathies and identification of therapies.
